# Cognitive development in children with chronic protein energy malnutrition

**DOI:** 10.1186/1744-9081-4-31

**Published:** 2008-07-24

**Authors:** Bhoomika R Kar, Shobini L Rao, B A Chandramouli

**Affiliations:** 1 Centre for Behavioural and Cognitive Sciences, Psychology Building, University of Allahabad, Allahabad, 211001, U.P., India; 2 Department of Clinical Psychology, National Institute of Mental Health and Neurosciences, PB No.: 2900, Hosur Road, Bangalore, 560029, Karnataka, India; 3 Department of Neurosurgery, National Institute of Mental Health and Neurosciences, PB No.: 2900, Hosur Road, Bangalore, 560029, Karnataka, India

## Abstract

**Background:**

Malnutrition is associated with both structural and functional pathology of the brain. A wide range of cognitive deficits has been reported in malnourished children. Effect of chronic protein energy malnutrition (PEM) causing stunting and wasting in children could also affect the ongoing development of higher cognitive processes during childhood (>5 years of age). The present study examined the effect of stunted growth on the rate of development of cognitive processes using neuropsychological measures.

**Methods:**

Twenty children identified as malnourished and twenty as adequately nourished in the age groups of 5–7 years and 8–10 years were examined. NIMHANS neuropsychological battery for children sensitive to the effects of brain dysfunction and age related improvement was employed. The battery consisted of tests of motor speed, attention, visuospatial ability, executive functions, comprehension and learning and memory

**Results:**

Development of cognitive processes appeared to be governed by both age and nutritional status. Malnourished children performed poor on tests of attention, working memory, learning and memory and visuospatial ability except on the test of motor speed and coordination. Age related improvement was not observed on tests of design fluency, working memory, visual construction, learning and memory in malnourished children. However, age related improvement was observed on tests of attention, visual perception, and verbal comprehension in malnourished children even though the performance was deficient as compared to the performance level of adequately nourished children.

**Conclusion:**

Chronic protein energy malnutrition (stunting) affects the ongoing development of higher cognitive processes during childhood years rather than merely showing a generalized cognitive impairment. Stunting could result in slowing in the age related improvement in certain and not all higher order cognitive processes and may also result in long lasting cognitive impairments.

## Background

Malnutrition is the consequence of a combination of inadequate intake of protein, carbohydrates, micronutrients and frequent infections [1]. In India malnutrition is rampant. WHO report states that for the years 1990–1997 52% of Indian children less than 5 years of age suffer from severe to moderate under nutrition [2]. About 35% of preschool children in sub-Saharan Africa are reported to be stunted [3]. Malnutrition is associated with both structural and functional pathology of the brain. Structurally malnutrition results in tissue damage, growth retardation, disorderly differentiation, reduction in synapses and synaptic neurotransmitters, delayed myelination and reduced overall development of dendritic arborization of the developing brain. There are deviations in the temporal sequences of brain maturation, which in turn disturb the formation of neuronal circuits [1]. Long term alterations in brain function have been reported which could be related to long lasting cognitive impairments associated with malnutrition [4].

A wide range of cognitive deficits has been observed in malnourished children in India. In a study, malnourished children were assessed on the Gessell's developmental schedule from 4 to 52 weeks of age. Children with grades II and III malnutrition had poor development in all areas of behaviour i.e., motor, adaptive, language and personal social [5]. Rural children studying in primary school between the ages of 6–8 years were assessed on measures of social maturity (Vineland social maturity scale), visuo-motor co-ordination (Bender gestalt test), and memory (free recall of words, pictures and objects). Malnutrition was associated with deficits of social competence, visuo-motor coordination and memory. Malnutrition had a greater effect on the immediate memory of boys as compared with those of girls. Malnourished boys had greater impairment of immediate memory for words, pictures and objects, while malnourished girls had greater impairment of immediate memory for only pictures. Delayed recall of words and pictures of malnourished boys was impaired. Malnourished girls had an impairment of delayed recall of only words. The same authors measured the intelligence of malnourished children using Malin's Indian adaptation of the Wechsler's intelligence scale for children. IQ scores decreased with the severity of malnutrition. Significant decreases were observed in performance IQ, as well as on the subtests of information and digit span among the verbal subtests [6]. The above study has shown that though there is decrease in full scale IQ, yet performance on all the subtests was not affected. This suggests that malnutrition may affect different neuropsychological functions to different degrees. Studies done in Africa and South America have focused on the effect of stunted growth on cognitive abilities using verbal intelligence tests based on assessment of reasoning [7]. Such an assessment does not provide a comprehensive and specific assessment of cognitive processes like attention, memory, executive functions, visuo-spatial functions, comprehension as conducted in the present study. Information about the functional status of specific cognitive processes has implications for developing a cognitive rehabilitation program for malnourished children.

A neuropsychological assessment would throw light on functional status of brain behaviour relationships affected by malnutrition. Deficits of cognitive, emotional and behavioural functioning are linked to structural abnormalities of different regions of the brain. Brain structures and brain circuits compute different components of cognitive processes [8]. Malnutrition has long lasting effects in the realm of cognition and behaviour, although the cognitive processes like executive functions have not been fully assessed [9].

The differential nature of cognitive deficits associated with malnutrition suggests that different areas of the brain are compromised to different degrees. A neuropsychological assessment would be able to delineate the pattern of brain dysfunction. Malnutrition is a grave problem in our country as 52% of our children are malnourished. Effects of protein-calorie malnutrition are inextricably blended with the effects of social cultural disadvantage; even within the disadvantaged class, literacy environment at home and parental expectation regarding children's education are powerful variables. Perhaps membership in a higher caste confers some advantage in regard to home literacy, and parental expectation. Short and tall children do differ in some cognitive tests, but not in all as demonstrated in a study done in Orissa, India [10]. But whether or not stunted growth alone is the causative variable for cognitive weakness is not determined as yet. Moreover, the functional integrity of specific cognitive processes is less clear. Chronic PEM resulting in stunting and wasting could result in delay in the development of cognitive processes or in permanent cognitive impairments. Neuropsychological measures can demonstrate delay in normally developing cognitive processes as well as permanent cognitive deficits.

The present study is an attempt to investigate the effect of stunting (as a result of PEM) on the nature of cognitive impairments and on the rate of cognitive development. Neuropsychological measures, standardized with respect to the age trends of cognitive processes in children in the age range of 5–15 years have been employed which would also inform about the neuropsychological performance of malnourished children. The study aimed to investigate if malnutrition would result in a diffuse impairment and a general slowing in the rate of development of all cognitive processes or these effects could be present for some specific cognitive processes. The purpose was also to determine the cognitive processes that are more vulnerable to the effects of malnutrition in two ways: in terms of impairment without affecting the rate of development and with respect to the effect on the rate of development of cognitive processes itself.

## Methods

### Participants

Children in the age range of 5–10 years attending a corporation school in the city of Bangalore participated in the study. Corporation schools in India are government schools with minimal fee attended by children from low-middle class. There were 20 children in adequately nourished group and 20 in the malnourished group. The gender distribution was equal. Children in both the groups were from the same ethnic/language background. They were natives of Karnataka living in Bangalore. Mother tongue of all the participants was Kannada. Informed consent was obtained from the parents. All the participants were screened for vision and color blindness using brief tests of visual acuity and color discrimination. Children with normal or corrected to normal vision were taken for the study. None of the participants had color blindness. All the children were right handed which was one of the inclusion criteria. Handedness was measured using the Edinburgh handedness inventory.

#### Malnourished group

Children studying in a corporation school were screened for malnutrition. The students of this school belonged to families belonging to the low socio economic status. Socio economic status was assessed by 1 the parental income as given in the school records. Chronic malnutrition was identified by anthropometric indices. These were height of the child for age (stunting) and body weight for the height (wasting) with reference to the national centre of health statistics (NCHS) standards of growth and development [11]. Stunting as well as stunting and wasting were taken as indices of moderate to severe malnutrition. Height for age and weight for height measures of each participant were compared to age and grade appropriate norms with reference to the NCHS standards of growth and development. Height for age/weight for height score less than 2 standard deviation (-2 SD) from the median was considered as indicative of moderate to severe malnutrition. In the age range of 5–10 years, children from preparatory to fifth grade were screened. Each grade had about 30 children. All the children in a class were taken for height and weight measurements. Out of the total of 180 children 14.4% children were found stunted or stunted and wasted. Only those children who were stunted or those who were both stunted and wasted were included in the malnourished group. Children who were only wasted were not included in the study to take account of only chronic PEM.

A semi-structured interview was used as a measure of home environment because a tool standardized on Indian population with norms based on population from south India was not available. The semi-structured interview was designed to collect information about the parents' educational level, recreational activities at home, caste. Mothers of most of the malnourished children were illiterate and fathers were literate. Watching television and playing indoors with friends were the only recreational activities at home. Children had the opportunity to play outdoors at school. Caste hierarchy did not differ much across children.

#### Adequately nourished group

Adequately nourished children were also taken from the same school in order to control the differences in socioeconomic background as well as home and school environment. It was important to take both the groups from the same school to control for factors like family background, literacy level of parents as the children attending the corporation school were mostly from similar locality and family backgrounds. Apart from this, the school environment, pattern of teaching was also similar for both the groups.

Adequately nourished children were matched with malnourished children with respect to age and grade level also. Anthropometric assessment was done for adequately nourished children also. Children who were ≥ 50^th ^percentile on the parameters of height for age and weight for height as per the NCHS standards were included in the study. Children in this group also came from families belonging to lower-middle as well as middle socioeconomic status groups similar to the malnourished group. The socio economic status was determined by parental income from the school records. Table 1 presents the details of the demographic characteristics of the participants.

**Table 1:** Demographic details of the participants

	Adequately nourished N = 20		Malnourished N = 20	
Mean age	5–7 years	8–10 years	5–7 years	8–10 years
	5.8 years	8.8 years	6.3 years	9.3 years
Gender	Girls:10	Boys: 10	Girls:10	Boys: 10
Stunted % (height for age -2 SD from the median)	----		70%	
Stunted and wasted % (height for age and weight for height: -2 SD from the median)	----		30%	

#### Exclusion of mental retardation

After identifying the malnourished and adequately nourished children the coloured progressive matrices test [12] was administered to rule out mental retardation. Children falling at or below the fifth percentile were excluded from the sample, as the 5^th ^percentile is suggestive of intellectually defective range. The percentile points were calculated from the raw scores using Indian norms [13]. Mental retardation was ruled out as otherwise scores on neuropsychological tests would be uniformly depressed and a differentiation of deficits might not occur. Intelligence was not treated as a covariate in the study. The groups did not differ significantly in their scores on CPM. CPM was taken as a screening instrument to rule out intellectual impairment in both the groups.

#### Exclusion of behaviour problems and history of neurological disorders

The children's behaviour questionnaire form B [14] was administered to the class teachers of the identified children. Children who scored above the cut off score of 9 were not included in the sample. The personal data sheet was filled in consultation with the parents and teachers to rule out any history of any neurological/psychiatric disorders including head injury and epilepsy and one child with epilepsy was excluded. This was one of the exclusion criteria.

Mean years of education for the adequately nourished group was 2.5 years for the younger children and 4.6 years for 8–10 years age group. Mean years of education for the malnourished group was 2.8 years for the younger children and 5.2 years for 8–10 years age group. F test did not find significant differences between the mean age of the malnourished and adequately nourished groups, which suggests that the two groups were matched. Mean years of schooling also did not significantly differ. Both the groups were taken from the corporation school in Bangalore city. A large percentage of children from the malnourished group as well as the adequately nourished group (92% and 89% respectively) came from low socio economic status.

### Instruments

#### NIMHANS neuropsychological battery for children [13]

NIMHANS neuropsychological battery for children was developed as a psychometric instrument for comprehensive neuropsychological assessment of children in the age range of 5–15 years. The battery consists of neuropsychological tests to assess motor speed, attention, executive functions, visuo-spatial relationships, comprehension, learning and memory. Instructions are in English as well as one local language that is Kannada. The participants in the present study were Kannada speaking children.

The battery has been standardized on a normative sample of 400 children (5–15 years). Norms were developed on the basis of empirical validation of age related differences using the growth curve modeling approach. Norms of the battery have been developed for each age level for 7 percentile points considering 5^th ^percentile as a cut off for accuracy measures and 95^th ^percentile for time measures. Cut-off scores for each test score and for each age level were calculated on the basis of the predicted values derived from the identified growth curves. The test retest reliability coefficients of the various tests in the battery range from .53 to .82 that indicates a fairly good test retest reliability of the battery. The battery has been validated on children with cortical tumors, intractable epilepsy and diffuse head injury. Norms of the battery were not used in the present study as we have grouped three age levels in each of the two age groups in the present study (5–7 and 8–10 years). Scores were also not compared to the age norms of the battery as the normative sample would not match the malnourished group on factors like family background, educational level of parents, socioeconomic status, school environment, and pattern of teaching.

The tests have been grouped under specific cognitive domains on the basis of theoretical rationale and factor analysis. Factor analysis has been done for the battery and the grouping of tests under cognitive functions like executive functions, visuospatial functions, comprehension and learning and memory was done on the basis of the clustering observed in factor analysis as well as on theoretical grounds.

The neuropsychological battery consisted of the following tests:

##### Motor speed

*1. Finger tapping test *[15]: This is a measure of motor speed. The test has shown stronger age effects than education in children and has been standardized on children in the west [16]. The subject is asked to tap the mounting key on a finger-tapping instrument as rapidly as possible using the index finger of the preferred hand. A comparable set of measurements is then obtained with the non-preferred hand. Five trials are given for each hand, with each trial lasting for 10 seconds. Average number of taps for each hand comprised the score. The finger tapping test has shown improvement in performance up to 9 years of age [17].

##### Expressive speech

2. Expressive speech test was administered to rule out speech related deficits. Expressive speech was assessed using tests of repetitive speech, nominative speech and narrative speech in question answer form. Purpose of this test was rule out speech related problems in the participants. None of the participants in both the groups showed speech related difficulties.

##### Attention

*3. Color trails test *[18] is a measure of focused attention and conceptual tracking. Children aged 5 to 16 years show a steady age progression on this test. It is sensitive to the effects of frontal lobe damage [19]. Children aged 8 to 16 years show a steady age progression on this test [20]. The participant was asked to serially connect the numbers 1–25 printed in two colours irrespective of the colour on colour trails 1. They were required to connect the numbers serially from 1 to 25 alternating between pink and yellow circles and disregarding the numbers in circles of the alternate color on colour trails 2. Time taken to complete each part is the score.

4. *Color cancellation test *[21] is a measure of visual scanning/selective attention. It consists of 150 circles in red, blue, yellow, black and grey. The participants were required to cancel only the yellow and red circles as fast as they can. Time taken in seconds to complete the test comprised the score.

##### Executive functions

5. *FAS phonemic fluency test *is a measure of *verbal fluency*. This test evaluates spontaneous production of words beginning with a given letter within a limited time. Deficits in verbal fluency have been found to be more in left frontal damage (70%) compared to right frontal damage (38%) [22]. Participants are asked to produce orally as many words as possible beginning with a given letter F, A, and then S. One minute is given for each letter. Words produced are noted. The test-retest reliability of the FAS test in 8 years old children is reported to be .54. Concurrent validity has also been established indicating better validity for letter fluency than for category fluency [23].

*6. Design fluency test *[24] is a measure of *design fluency*, cognitive flexibility and imaginative capacity. It is a visual analogue of verbal fluency task. Patients with right frontal or central damage have difficulty on this test [25]. The participants are required to generate and draw as many abstract designs as possible in five minutes. The participants obtain a novel output score and a perseverative score. Children show improvement in scores on design fluency test until 12 years of age [17].

*7. Visuo-spatial working memory span task *[23]: This test is a measure of visuo-spatial working memory (VSWM) span. A gradual improvement in working memory from childhood until adolescence has been reported [26]. Another study showed a steady linear improvement in performance between 5–12 years on the VSWM span task [27]. A developmental study on children across 5–13 years of age showed a steady developmental trend between 5–8 years with respect to the maintenance and manipulation components of VSWM [28].

The visuospatial working memory span task consists of 4 cubes/blocks arranged in a row. The examiner taps these four cubes with a fifth cube. The tapping is performed in different sequences as given below. The subject is required to repeat the sequence of four taps tapped by the examiner. Five trials of forward and five trials of reverse sequence are given. Number of taps remains the same for all the forward and reverse sequences but each sequence is different from the other. Number of correct sequences tapped by the subject for both the forward and reverse condition together comprises the total score for this test.

##### Visuospatial functions

*8. Motor-free visual perception test *[29] is a measure of *visuoperceptual ability*, having 36 items for visual discrimination, visual closure, figure-ground, perceptual matching and visual memory. Since this test has been originally developed for children between 5–8 years of age, it was modified and items in increasing difficulty level were added by the authors to make it applicable for the children above 8 years. Number of correct responses comprises the score.

*9. Picture completion test *[30] is a measure of visuoconceptual ability, visual organization and visuo-conceptual reasoning. It consists of 20 cards with pictures of different objects with a missing feature. The participants are required to name or point out to the missing feature. Number of correct responses comprises the score.

*10. Block design test *[30] is a measure of visuoconstructive ability. It consists of 10 designs to be constructed using 4–9 blocks in a specified time limit. Performance on this test is scored with respect to the time taken to complete each item.

##### Comprehension, learning and memory

*11. Token test *[31] is a measure of verbal comprehension of commands of increasing complexity. It is a sensitive test of receptive aphasia and also developmental aphasia. Its sensitivity has been reported by demonstrating problems of speech comprehension by patients who showed no difficulty in understanding a normal conversation. It consists of tokens in two shapes (circle and square), two sizes (large and small) and 5 colours (red, blue, yellow, green, and white). There are 36 commands read out one by one by the examiner. As the examiner gives the command, the subject has to carry it out by manipulating the tokens accordingly. One point is given for each correctly performed item. A correct response after one repetition earns a score of 0.5. Two repetitions are deemed as failure.

*12. Rey's auditory verbal learning test (RAVLT) *[32] is a measure of verbal learning and memory. It is a measure of immediate memory, acquisition or new learning, retention, primacy, and recency effect, susceptibility to proactive and retroactive interference. Nonlinear age effects on RAVLT have been reported in children, with greater improvement in performance during middle childhood than during early adolescence [33]. Age related improvement up till 9 years of age have been reported on RAVLT [17]. It consists of a list of 15 words presented five times with an immediate recall after each of the 5 trials. A delayed recall is taken after a delay of 30 minutes filled with other nonverbal tests.

*13. Memory for designs test *[34] is a measure of visual learning and memory. The role of right temporal lobe in memory for visual patterns is well documented [35]. The test consists of 18 abstract designs each printed on a separate card. Number of designs presented to the children varies across age groups. Five learning trials are given with a delayed recall after a delay of one hour filled with verbal tests. Number of correct designs on each of the five trials gives the learning rate. Number of correctly recalled designs on delayed recall is the other score.

### Procedure

All the neuropsychological tests were individually administered to the children in well-controlled and testable conditions. Two rest pauses of five minutes each were given to avoid the effect of fatigue on test performance. All the tests had accuracy as the measure of performance except the color cancellation test and color trials test in which the measure of performance was time in seconds. Color trails test showed a significantly poor performance in malnourished children but it was not taken as a variable while comparing the age related differences between the two age groups as this test was not administered for 5–7 years age group. The battery on average took two and a half hours to administer. Some of the tests like verbal fluency and design fluency being timed tests take 3–4 minutes only. Some malnourished children were slow in responding so it took a litter more time for them. Adequate rest pauses were given during the assessment. Light refreshment was offered during the break other than the two five minute rest pauses.

## Results

### Statistical analysis

ANOVA was computed to compare the performance of the two groups of children across the two age groups (Table 2). Statistical package for social sciences (SPSS 10) was used for statistical analysis. Post hoc comparisons for each test score were computed using the Tukey's post-hoc test.

**Table 2:** Mean comparisons for the cognitive functions across the two age groups of adequately nourished and malnourished children

		**WN **(n = 20)		**MN **(n = 20)		
		
**Functions**	**Test scores**	Mean (SD)		Mean (SD)		**F ratio**
			
		5–7 yrs	8–10 yrs	5–7 yrs	8–10 yrs	
**Motor functions (primarily mediated by frontal cortex)**						

Motor speed-Right hand	Finger tapping test: Avg no. of taps	26.5 (8.6)	27.9 (4.5)	24.7 (2.2)	28.3 (3.2)	1.63ns
Motor speed-Left hand	Finger tapping test: Avg no. of taps	26.1 (9.1)	26.2 (5.08)	21.5 (2.8)	24.9 (1.51)	.85ns

**Attention (primarily mediated by frontal and parietal cortex)**						

Selective attention	+Colour cancellation test	129" (20.2)	95.9" (18.3)	202.8" (17.7)	153.6" (19.6)	9.4**
Focused attention	Colour trails test +Trail A	---	105" (25.8)	---	249" (49.2)	4.09**
	+Trail B	---	213.4" (18.1)	---	383.6" (27.5)	4.06**

**Executive functions (primarily mediated by dorsolateral prefrontal cortex)**						

Verbal fluency	FAS phonemic fluency test	4.3 (1.36)	5.7 (1.8)	1.36 (.15)	4.4 (2.3)	10.8**
Design fluency	Design fluency test	5.4 (2.4)	10.3 (1.06)	3.2 (1.94)	4.5 (2.4)	7.75**
Working memory	Visuospatial working memory span task	5.6 (2.02)	7.6 (2.05)	3.2 (1.2)	4.2 (2.4)	7.4**

**Visuospatial functions (primarily mediated by right parietal cortex)**						

Visual perception	Motor free visual perception test	26 (2.7)	30 (2.4)	17.4 (2.03)	20.4 (3.8)	7.8**
Visual construction	Block design test	10 (2.6)	15.8 (5.5)	3.0 (1.3)	4.8 (2.2)	9.37**
Visual conceptual reasoning	Picture completion test	9.4 (1.8)	10 (1.3)	6.0 (2.0)	7.0 (2.7)	7.87**

**Comprehension, learning and memory (primarily mediated by bilateral medial and anterior lateral temporal cortex)**						

Verbal comprehension	Token test	27.7 (2.0)	30.6 (1.5)	19.6 (4.4)	23 (5.4)	14.2**
Verbal learning	Rey's auditory verbal learning test (RAVLT)-total learning score	32.4 (11.8)	42.3 (11.7)	26.9 (11.0)	30.7 (9.3)	4.2*
Verbal memory	Delayed recall score, RAVLT	7.1 (2.2)	8.9 (1.7)	5.6 (2.01)	6.0 (1.8)	5.08*
Visual memory	Delayed recall score, memory for designs test	6.6 (1.6)	9.7 (3.5)	2.2 (1.8)	2.4 (1.6)	8.04**

* p < .05; **p < .01; ns p > .05; +: time in seconds; WN: adequately nourished, MN: malnourished

### Comparison between the performance of adequately nourished children and malnourished children

Table 2.0 shows that malnourished group differed significantly from the adequately nourished group on tests of phonemic fluency, design fluency, selective attention, visuospatial working memory, visuospatial functions, verbal comprehension and verbal learning and memory showing poor performance. The two groups did not differ on the test of finger tapping. Since expressive speech was a question answer type assessment looking at repetitive speech, nominative speech and narrative speech, which is like an initial screening for aphasia, like symptoms. Since it did not give a quantitative score, hence was not taken for analysis. As a descriptive account of expressive speech it was observed that malnourished children did not have any difficulty with respect to expressive speech.

### Comparison of age related differences in cognitive functions between adequately nourished and malnourished children

**Figure 1:**
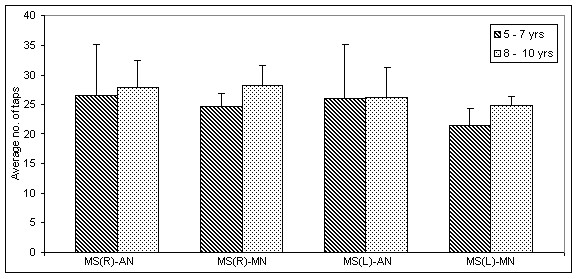
**Age related comparisons between adequately nourished and malnourished children on motor speed (right and left hand)**. Note: MS (R): motor speed (right hand); MS (L): motor speed (left hand); AN: adequately nourished; MN: malnourished.

**Figure 2:**
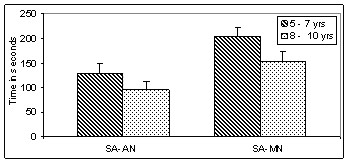
**Age related comparisons between adequately nourished and malnourished children on selective attention (color cancellation test)**. Note: SA: selective attention; AN: adequately nourished; MN: malnourished.

**Figure 3:**
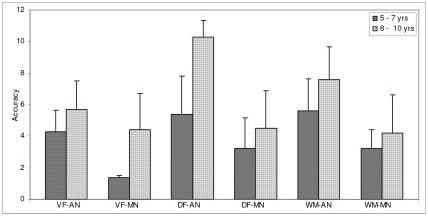
**Age related comparisons between adequately nourished and malnourished children on executive functions**. Note: VF: verbal fluency; DF: design fluency; WM: working memory; AN: adequately nourished; MN: malnourished.

**Figure 4:**
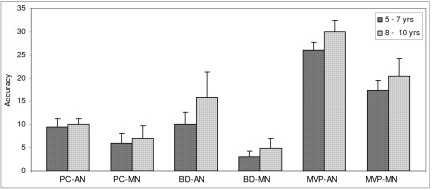
**Age related comparisons between adequately nourished and malnourished children on visuospatial functions**. Note: PC: picture completion; BD: block design; MVP: motorfree visual perception; AN: adequately nourished; MN: malnourished.

**Figure 5:**
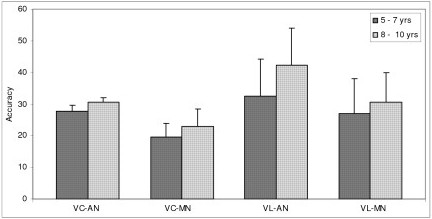
**Age related comparisons between adequately nourished and malnourished children on verbal comprehension and verbal learning**. Note: VC: verbal comprehension; VL: verbal learning; AN: adequately nourished; MN: malnourished.

**Figure 6:**
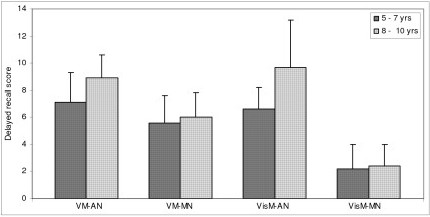
**Age related comparisons between adequately nourished and malnourished children on verbal and visual memory**. Note: VM: verbal memory; VisM: visual memory; AN: adequately nourished; MN: malnourished

Data was further subjected to post hoc analysis to compare the two groups across the two age groups to study the rate of improvement with age (Table 2). In both the age groups of 5–7 years and 8–10 years the adequately nourished children performed better than the malnourished children. Figures 1, 2, 3, 4, 5, 6 indicate age related improvement in performance across different cognitive functions in adequately nourished children as compared to malnourished children. Motor speed and coordination was not significantly affected in malnourished children as compared to the adequately nourished children (figure 1). The rate of age related improvement across the two age groups was found rapid on certain functions like selective attention (figure 2) and verbal fluency (figure 3) in malnourished children. However, working memory, design fluency, visuospatial functions, comprehension, learning, and memory showed slowing in terms of age related improvement in malnourished children. Most of the cognitive functions like design fluency (figure 3), working memory (figure 3), Visual perception (figure 4), visuo-conceptual reasoning (figure 4), visual construction (figure 4), verbal comprehension (figure 5), verbal and visual memory (figures 6) have shown a very slow rate of improvement with respect to the difference in performance between the two age groups of 5–7 and 8–10 years. On the contrary functions like verbal fluency (figure 3), motor speed (figures 1), and selective attention (figure 2) showed similar rates of improvement in adequately nourished children and malnourished children while comparing the two age groups.

Post-hoc comparisons were computed with Tukey's post-hoc tests to compare the means across age groups between malnourished and adequately nourished children for those test scores that showed significant effects. Hence, post hoc tests were not computed for the finger tapping test scores assessing motor speed. Table 3 presents the post-hoc results with the significance (probability level) levels of the differences across age groups and between adequately nourished and malnourished children.

**Table 3:** Post-hoc comparisons between adequately nourished and malnourished groups across the two age groups

**Groups**	**Variables**	**Probability level**
**Selective Attention **(color cancellation test: time in seconds)		

AN	5–7 vs 8–10	p < .05
MN	5–7 vs 8–10	p < .05
5–7 years	AN vs MN	p < .05
8–10 years	AN vs MN	p < .05

**Executive functions**		

Cognitive flexibility (FAS phonemic fluency test)		
AN	5–7 vs 8–10	p > .05
MN	5–7 vs 8–10	p < .05
5–7 years	AN vs MN	p < .05
8–10 years	AN vs MN	p > .05
Cognitive flexibility (design fluency test)		
AN	5–7 vs 8–10	p < .05
MN	5–7 vs 8–10	p > .05
5–7 years	AN vs MN	p < .05
8–10 years	AN vs MN	p < .05
Working memory (tapping blocks in sequence)		
AN	5–7 vs 8–10	p > .05
MN	5–7 vs 8–10	p > .05
5–7 years	AN vs MN	p < .05
8–10 years	AN vs MN	p < .05

**Visuospatial functions**		

Visual perception (motor-free visual perception test)		
AN	5–7 vs 8–10	p > .05
MN	5–7 vs 8–10	p > .05
5–7 years	AN vs MN	p < .05
8–10 years	AN vs MN	p < .05
Visuo-conceptual reasoning (picture completion test)		
AN	5–7 vs 8–10	p > .05
MN	5–7 vs 8–10	p > .05
5–7 years	AN vs MN	p < .05
8–10 years	AN vs MN	p > .05
Visuoconstructive ability (block design test)		
AN	5–7 vs 8–10	p < .05
MN	5–7 vs 8–10	p > .05
5–7 years	AN vs MN	p < .05
8–10 years	AN vs MN	p < .05

**Comprehension, learning and memory**		

Verbal comprehension (token test)		
AN	5–7 vs 8–10	p > .05
MN	5–7 vs 8–10	p > .05
5–7 years	AN vs MN	p < .05
8–10 years	AN vs MN	p > .05
Verbal learning (RAVLT)		
AN	5–7 vs 8–10	p < .05
MN	5–7 vs 8–10	p > .05
5–7 years	AN vs MN	p > .05
8–10 years	AN vs MN	p < .05
Verbal memory (RAVLT)		
AN	5–7 vs 8–10	p > .05
MN	5–7 vs 8–10	p > .05
5–7 years	AN vs MN	p > .05
8–10 years	AN vs MN	p < .05
Visual memory (memory for designs test)		
AN	5–7 vs 8–10	p > .05
MN	5–7 vs 8–10	p > .05
5–7 years	AN vs MN	p < .05
8–10 years	AN vs MN	p < .05

Note: AN: adequately nourished; MN: malnourished.

Post hoc results have been done to support our theoretical claims about the lack of age related improvement in certain cognitive functions on one hand and the nature of cognitive impairments on the other in malnourished children. Four comparisons were interpreted i.e., comparing performance between the two age groups of adequately nourished and malnourished children separately. The other comparison was between the adequately nourished and malnourished children for the age group of 5–7 years and similarly for the age group of 8–10 years. Results indicate age related differences within each group as well as between the two groups. Age related differences were found significant for some of the test scores between 5–7 and 8–10 year old children in the adequately nourished group but not for most of the test scores for malnourished group indicative of a delay in development of certain cognitive functions. Differences were found significant between the adequately nourished and malnourished children for the same age group for most of the test scores indicative of a deficit in a particular cognitive function. In few of the tests, performance was not found to be significantly different between the two age groups for both adequately nourished and malnourished children.

Results indicate a lack of age related improvement in malnourished children with respect to cognitive functions of attention, cognitive flexibility, visuo-constructive ability and verbal learning. Post-hoc results also indicate cognitive impairments in attention, working memory, visuo-perceptual ability, comprehension, and visual memory, revealed by significantly poor performance of malnourished children as compared to adequately nourished children in the same age group. Lack of age related improvement in malnourished children was not observed for functions like working memory, visuo-perceptual ability, comprehension, and memory. For these functions malnourished showed poorer performance as compared to adequately nourished children although they showed an age related improvement for the same functions. This is indicated by a non-significant difference between the two age groups for adequately nourished as well as malnourished children. However, the performance was impaired for these functions as indicated by the significant differences between adequately nourished and malnourished for each age group. These results need verification with a larger sample but they show interesting trends about the vulnerability of cognitive functions being affected by malnutrition (stunting).

## Discussion

The findings of the present study could be discussed in terms of the effect of chronic malnutrition on neuropsychological performance and with respect to the rate of development of cognitive processes.

### Effect of malnutrition on neuropsychological performance

Our study indicates that malnourished children perform poor on most of the neuropsychological tests except that of motor speed as compared to adequately nourished children. Malnourished children showed poor performance on tests of higher cognitive functions like cognitive flexibility, attention, working memory, visual perception, verbal comprehension, and memory. These findings are supported by another study on Indian malnourished children, which reported memory impairments in undernourished children and spared fine motor coordination [36]. Malnourished children showed poor performance on novel tasks like tests of executive functions i.e., working memory spatial locations. Poor performance on the tests of fluency and working memory also coincides with very slow rate of improvement between the age groups of 5–7 years and 8–10 years. Poor performance on most of the neuropsychological tests indicated a diffuse impairment including attention, executive functions, visuospatial functions, comprehension and memory.

### Effect of malnutrition on cognitive development

Both the groups were tested on a neuropsychological battery, which has been found to be sensitive to age related differences in cognitive functions in children (5–15 years). The age trends reported in the present study are based on the assessment that employed the NIMHANS neuropsychological battery for children [13]. The test battery has been standardized based on the growth curve modeling approach for empirical validation of age-related differences in performance on neuropsychological tests. The tests in the battery were found sensitive to show age related differences.

Malnourished children showed poor performance with respect to age as compared to adequately nourished children. The performance of malnourished children in the 5–7 years age group was poor and much lower than the adequately nourished children and did not seem to show much improvement in the 8–10 years age group. The rate of cognitive development was found to be different for different cognitive functions. The rate of development was affected for some of the cognitive functions showing minimal age related improvement across the age range of 5–7 years and 8–10 years such as design fluency, working memory, visual construction, verbal comprehension, learning and memory for verbal and visual material. On the contrary, age related improvement was observed on certain other cognitive functions in malnourished children, where the level of performance was low for both the age groups but the rate of improvement between the two age groups was similar to adequately nourished children.

*Motor speed *(right and left hand) was not found impaired in malnourished children and the rate of development was also found similar to adequately nourished children. Chronic protein energy malnutrition does not seem to affect basic cognitive processes like motor speed which is affected in case of other nutritional deficiencies.

*Executive functions *such as design fluency, selective attention and working memory were found deficient in malnourished children also showing poor rate of improvement between the two age groups. All the three tests of executive functions like fluency, selective attention and working memory for spatial locations involved novel stimuli and performance required cognitive flexibility as well as faster information processing which was affected in malnourished children. Results also indicate that malnourished children showed a very slow rate of improvement on these functions. At one level age appropriate performance on tests of executive functions is affected as well as the gain due to increase in age is also affected. However, in case of verbal fluency the performance of malnourished children was poor but the rate of improvement between the two age groups was better than adequately nourished children.

*Visuo-spatial functions *like visual perception, visual construction and visuo-conceptual reasoning showed significantly poor performance when compared to the adequately nourished children but showed a steep age related improvement in performance. Performance on functions like visual perception (visual discrimination, perceptual matching, visual closure and visuospatial relationships) and visual construction was severely affected in malnourished children and also showed poor rate of improvement with age.

*Verbal comprehension, learning and memory for verbal and visual material* was found poor as compared to adequately nourished children but the rate of improvement between 5–7 years age group and 8–10 years age group was similar to that of adequately nourished children.  These results suggest that development of comprehension with age might not be affected in malnourished children. However, other than the poor performance on the AVLT test of verbal learning, malnourished children also showed minimal improvement between the two age groups as compared to the greater magnitude of difference between the two age groups in adequately nourished children. Visual memory was most severely affected in malnourished children in terms of the poor performance on delayed recall on design learning test as well as in terms of the difference between the two age groups.

Malnutrition affects brain growth and development and hence future behavioral outcomes [37]. School-age children who suffered from early childhood malnutrition have generally been found to have poorer IQ levels, cognitive function, school achievement and greater behavioral problems than matched controls and, to a lesser extent, siblings. The disadvantages last at least until adolescence. There is no consistent evidence of a specific cognitive deficit [38]. The functional integrity of specific cognitive processes is less clear.

Stunting in early childhood is common in developing countries and is associated with poorer cognition and school achievement in later childhood [39]. Deficits in children's scores have been reported to be smaller at age 11 years than at age 8 years in a longitudinal study on malnourished children stunted children suggesting that adverse effects may decline over time [7]. In our study also all the children in malnourished group were stunted and the cross sectional assessment of age related improvement has shown similar rate of improvement across 5–7 years to 8–10 years age groups as observed in adequately nourished children though the baseline performance was low in malnourished children. These results indicate that the adverse effects of malnutrition (stunting in particular) may decline with age only for certain cognitive functions but the rate of cognitive development for most of the cognitive processes particularly higher cognitive processes including executive processes and visuospatial perception could be severely affected during the childhood years. Decline in the effects of malnutrition overtime has been reported to be independent of differences in educational, socioeconomic and psychosocial resources [7]. Hence, malnutrition (particularly stunting) may result in delayed development of cognitive processes during childhood years rather than a permanent generalized cognitive impairment.

The neuropsychological interpretation of the cognitive processes more severely affected in malnourished children suggests a diffuse cortical involvement. This is with reference to deficits pertaining to functions mediated by dorsolateral prefrontal cortex (poor performance on tests of attention, fluency and working memory), right parietal (poor performance on tests of visuospatial functions) and bilateral temporal cortex (poor performance on tests of comprehension, verbal learning, and memory for verbal and visual material). The prefrontal cortex may be particularly vulnerable to malnutrition [4]. The adverse effects of malnutrition (PEM-stunting) on cognitive development could be related to the delay in certain processes of structural and functional maturation like delayed myelination and reduced overall development of dendritic arborization of the developing brain [1].

The present study highlights two ways in which malnutrition particularly stunting could affect cognitive functions. On one hand age related improvement in cognitive performance is compromised and on the other hand there could be long lasting cognitive impairments as well. However, the effect is nor specific to a particular cognitive domain and is rather more diffuse. Results of the study also indicate that: certain cognitive functions could be vulnerable to the effect of malnutrition in terms of showing impairment but the rate of development of these functions may not be affected. On the other hand, rate of development of certain cognitive functions may be affected and may also show impairment when compared with adequately nourished children.

## Conclusion

Chronic protein energy malnutrition (stunting) results in cognitive impairments as well as slowing in the rate of the development of cognitive processes. Rate of development of cognitive functions may follow different patterns in children with malnutrition. Chronic protein energy malnutrition affects the development of cognitive processes differently during childhood years rather than merely showing an overall cognitive dysfunction as compared to adequately nourished children. Stunting could result in delay in the development of cognitive functions as well as in permanent cognitive impairments which show minimal improvement with increase in age. Rate of development of attention, executive functions like cognitive flexibility, working memory, visuospatial functions like visual construction is more severely affected by protein energy malnutrition in childhood years, a period that is marked by rapid ongoing development of cognitive functions.

## Competing interests

The authors declare that they have no competing interests.

## Authors' contributions

BRK has made substantial contribution to the conception, design, data collection and analysis for the study. SLR and BAC have contributed to the conception, design and in drafting the manuscript. All authors read and approved the final manuscript.
